# Sour Fruits on the Trail: Renewing Phenomenological Practice

**DOI:** 10.5964/ejop.v11i3.1035

**Published:** 2015-08-20

**Authors:** Roberta De Monticelli, Andrei Simionescu-Panait

**Affiliations:** a Vita-Salute San Raffaele University, Milan, Italy; b Faculty of Philosophy, University of Bucharest, Bucharest, Romania

**Keywords:** Italian phenomenology, Husserl, emotions and values, Schwarze Hefte, applied phenomenology and politics

## Abstract

This summer, Europe’s Journal of Psychology hosts a fruitful discussion about phenomenology, its method, the possibilities of application in today's context and its current troubled waters stemming from recent historical-ideological debates. Prof. Roberta De Monticelli offers lush and informative answers to provocative issues like overdriving the epoché, Heidegger's dark undertones, the relation between pedagogy and authorship in phenomenology and the idea of filtering politics through Husserlian phenomenology.

**Andrei Simionescu-Panait:** Each year, the San Raffaele University hosts an international Spring School, which gathers around for debate numerous big authors in philosophy and connected areas of research, as well as plenty of students. This year, the event (“Joint Commitment. Collective Intentionality, Trust and Political Obligation”) hosted a direct dialogue between phenomenologists, experts in social ontology, applied ethics, normativity and researchers interested by intersubjectivity and empathy – in short, an important interdisciplinary event. How would you appreciate the presence of Husserlian concepts or Schelerian axiology in this fresh environment of ideas? Does phenomenology display a tendency of growth in recognised importance, considering research in recent years?

**Roberta De Monticelli:** The latter question is important. Indeed, we are looking at a re-awakening of true phenomenological research all over the world. This rekindled interest in all sorts of issues (De Monticelli, 2013) related to what Lynne Baker would call “the metaphysics of everyday world” (Baker, 2007) is the exciting fact of the last years, along with a more and more widespread involvement of trained philosophers in the research projects of a variety of sciences, addressing the natural and the social aspects of human life. A lot of water has flowed under the bridge since that harsh judgment made by Thomas Metzinger, according to whom phenomenology was “a discredited research program… intellectually bankrupt for at least 50 years” (Gallagher & Zahavi, 2008). Shaun Gallagher and Dan Zahavi, who are among the protagonists of the phenomenological renaissance at the very heart of contemporary philosophy of mind, point out three developments in cognitive science, which brought about a possible rehabilitation of phenomenology: 1. A revived interest in phenomenal consciousness; 2. The advent of embodied approaches to cognition; 3. The amazing progress in neuroscience and – I would add – the increasing need for accurate description of life-world phenomena whose neurobiological correlates one wants to investigate.

[Contrib contnr1]Now we can come back to the first part of your question. More and more scholars and scientists all over the world are getting familiar with that style of research that has been termed as the “4 *e* phenomenology”. Its rationale is the insight that no satisfactory account of the mind can be offered without considering mind’s *embodiment*, the environment in which a living body is *embedded*, the way in which life is *enacted* within such a life-world, and the extra-bodily *extensions* of mind making up the cultural layer of a human, and indeed social, life-world. That interdisciplinarity you mention is somehow built into the very method of any serious phenomenological research, because of the very richness of intertwined contents making up even the simplest bit of reality, as it is given to our consciousness. We devoted this year’s Spring School to such a pervasive phenomenon as the sort of bond which ties together the agents of any common enterprise, from a simple walk to a concert, from the ordinary working of a Department of philosophy to the destiny of a nation, or of Europe. *Joint commitment* is the title of an admirable (and disputable) book by Margaret Gilbert, but it’s also a key concept pinning down a source of social normativity. As most concepts in contemporary analytic philosophy, it is both a guideline for conceptual analysis of complex facts, and a template needing intuitive fulfilment, that is, requiring or at least exciting phenomenological descriptions and insights. Is a conjugal pact, or even a friendship, a bond, and a source of deontology, of exactly the same nature of, say, a commercial partnership? What distinguishes the three latter joint commitments from the one underlying a quarrel, or even a refined philosophical discussion? As soon as one raises these questions, all the fine-grained phenomenological descriptions of different “forms” of collective intentionality, from basic pre-personal collective habits up to sharing values of a personal level, come to the mind, and one sees why Moritz Geiger, a phenomenologist of the Munich circle, called phenomenology “a passion for distinctions”. An ideal travel mate for some bold generalizing minds, issued from the analytic tradition, such as most keynote speakers of our past Schools: such as Peter van Inwagen on Free Will, Lynne Baker on Personhood, Barry Smith on Taste, John Searle on Status Functions or Margaret Gilbert herself on Joint Commitment. Thus, your reference to Husserlian concepts or Schelerian axiology is quite to the point. *Gemeingeist* is the title of one of the manuscripts out of which *Ideas II* should have been worked out in one of the phases of its vexed history. Also, shared values are one of the main issues of Scheler’s two masterpieces, *Formalism in Ethics* and *On the Nature and Forms of Sympathy*.

**Andrei Simionescu-Panait:** There's an academic anathema floating on Heidegger's head, with all the *Schwartze Hefte* English translation and related discussions. The popular conception that places Heidegger as a philosopher who bettered Husserlian phenomenology is now fading. As Jeanne Hersch remarked, he had the chance of either being “the first priest of a new liturgy, or the last priest of a forgotten one” (Hersch, 1936), by the way of his signature “back to the Greeks” rally call. Meanwhile, Husserlian phenomenologists carry on with their work, while others admit radical changes of heart towards Heidegger (Figal & Schulte, 2015), at a time when the separation between a philosopher's concepts and his life carries no relevance in argumentation. Also, it is no secret that young phenomenologists worldwide turn to Husserl, Scheler or Merleau-Ponty as starting points for their future projects. Can these events play an important role in bridging the (still active) analytic/continental divide?

[Contrib contnr2]**Roberta De Monticelli:** You yourself quoted, in your third-last question, a relevant passage in a letter by Husserl, addressed to Alexander Pfaender, on Husserl’s bewilderment about “the gross misunderstanding” that he himself could have anything to do with “a philosophical system of the kind which I have always considered it my life's work to make forever impossible”. We shall come back to that issue. What is really important now is focusing on the very horror of Martin Heidegger’s thoughts recently published with the *Schwarzen Heften*. That they should be published, was part of Heidegger’s explicit will: they belong to his legacy to posterity. And, thank God, posterity has taken a stand, albeit with a lot of stunning exceptions. Some persist in ignoring the deep link between Heidegger “dictatorial and irresponsible way of thinking” (Hersch, 1988) and his wholehearted public support of the Nazi power. Jeanne Hersch, whose chair of Modern and Contemporary Philosophy I took over in Geneva (1989-2004) has been one of my masters. She has been my “continental” guide into the best of European practical philosophy, apart from the phenomenological legacy which I had learnt at my mother's knee so to speak, at the State University of Milan, even before starting my study of philosophy at the Scuola Normale in Pisa. Now what I learned from her, with astonishment, is that the worst of implications from Heidegger’s thought were crystal clear for the ones who had really got to know him and his teaching in the Thirties. She was among the students of Heidegger in Freiburg, along with Hannah Arendt and, above all, Raymond Klibansky. Klibansky became a great historian of the Platonic tradition: he was the youngest member of the Warburg circle and the one who rescued the Warburg library from the Nazi officials by adventurously transferring it to London, just in time to help himself to safety as well. He has been another of my “continental” masters: I had met him in Oxford, where I spent a year as a doctoral student long before being appointed at Geneva University. Both of them, Hersch and Klibansky, Jews of Polish origin, had also been students of Karl Jaspers in Heidelberg (like Hannah Arendt) in those fatal years, the early thirties, during which Heidegger holds the Rectorship speeches (more than one, indeed) and entered the NS party (which he did not quit any more, nor did he ever publicly take any distance from any of his Nazi engagements). What was crystal clear to Jeanne Hersch and Raymond Klibansky (and to Karl Löwith as well, albeit to a lesser degree of clarity) – are the very *theoretical* roots, in Heidegger’s system of thought, of an endorsement to any possible anti-humanistic, illiberal and in fact violent political adventure. Even the discovery of such obscene passages, in the *Schwarzen Heften*, as the ones charging the Jews with their own extermination, in their role of “mouthpiece of Modernity” and hence of “objectification” of Being, technology and disenchanted desire for power, did not feel surprising then. Emmanuel Faye’s research has added more evidence and more philosophical analysis to that of Victor Farias, but the big question, in the light of Klibansky’s and Hersch’ writings, is still the same: how was it possible for such a long time that a vast majority of leading philosophers, especially in France and Italy, have been so utterly indifferent to the radical *destruction of the practical reason,* this actual legacy of Enlightenment and Socraticism, hence of “European” philosophy to court, that the “Hirsch of Being” had been preaching in his pompous and sophistic language?

Our PhenomenologyLab hosted a rich discussion on those issues, on the occasion of a conference held this year at San Raffaele University on the *Schwarze Hefte*. I myself resumed the theses I had been defending for more than twenty years on the sophistic core of Heidegger’s thought, destroying the very norms of logical argument and ethical judgement^i^.

But let’s turn to a related issue which is more to the point of your question. The reason why many of the researchers and scholars who are currently placed within the *phenomenological* tradition did ignore the dark side of Heidegger’s thought (starting from Sartre, alas) is another astonishing fact, haunting almost the whole history of the scholarly research *on* Husserl and phenomenology (which I would keep quite distinct from research *in* phenomenology; Spiegelberg, 1975). My claim about this fact is that it causally depends on a most surprising neglect of the living and the vivid *practical* soul of phenomenology. By “practical” I mean “ethical” in the broad sense in which Husserl used to employ this word, both at the very beginning of his philosophical career and in his numerous, systematic courses on Ethics. Practical reason is essentially “intertwined” with “theoretical” reason, as logics is not really “parallel”, but “intertwined” with ethics (Husserl, 1908-1914). The very heart of Husserl's thought beats in his question on the sources of normativity, a question he meant in so radical a way as to invest logical, axiological and practical normativity alike. Husserl’s answer provides a basis for the *pars construens* of the *Logical Investigations,* starting from that masterpiece analysis in *Prolegomena* §14, which displays the equivalences between eidetic and axiologic statements and their normative or deontic versions. It is impossible to see the unitary project being developed throughout the six *Investigations* without considering this main claim about the eidetic sources of normativity, overarching all detailed inquiries into the given eidetic (ontological) bonds “keeping together”, within definite ranges of possible variations, all parts and moments of any concrete object. This *pars construens* of the phenomenological thought, i.e. the discovery and exact conceptualization of what I proposed to call “The gift of bonds” (De Monticelli, 2013b, 2013c), underlies its *pars destruens* as well, namely the two reductions to absurdity of Scientific Naturalism on one hand (quite particularly in its application to Mind, i.e. “Psychologism”) and Historicism/Cultural Relativism, on the other hand.

To sum up, Husserlian phenomenology involves a most innovatory and bold project of a rational foundation of practical thought, i.e., the kind of thought which can enlighten and direct our actions, in the wide sense of “action” in which we meaningfully enact our lives in the life – and social worlds where humans meet. In this wider sense, cognition too is action, and so is experience. No cognition, no experience is possible without some standard of correctness, i.e. without normativity. A criticism of pure and practical reason, questioning and rejecting the main tenet of Kantianism, i.e. the gaps between data and apriori, experiences and norms, phenomena and noumena, cognition and duty, resuming the reject of all these dualisms by the very notion of *material apriori*: such is Husserlian phenomenology in the eye of his founder himself (Husserl, 1956b). Such a phenomenology almost emphatically calls for a material axiology, a complementary achievement of practical reason, which is to a formal axiology what the material ontology of the ontological “regions” is to formal ontology. Husserl’s thought calls for Scheler’s, a formal ethics craves to be implemented by a “material” ethics of values, as the one that Scheler provided with his masterpiece (Scheler, 1973).

Now a phenomenology so conceived is utterly incompatible with a system of thought rejecting the very tenets of socraticism and humanistic, enlightened reason, as the one represented by Heidegger and his followers. I am not sure that even the best contemporary phenomenologists are fully aware of this, and I wonder whether such an awareness is common enough among young phenomenologists worldwide who, as you said, turn to Husserl, Scheler or Merleau-Ponty as starting points for their future projects. There’s more than a methodological divide in the (in itself absurd) opposition between a continent and a method, which is the analytic/continental divide. It is no chance that this divide did not really exist before breaking out with Carnap’s bitter (and so reasonable, in spite of Carnap’s own part of empiricist dogmatism) pamphlet against Heidegger’s best seller, *Was ist Metaphysik* (Carnap, 1931). It did not exist at a time where Husserl and Frege and Russell read each other, Moore read Brentano and Austin quoted Husserl. It wasn’t that dramatic either, in the world where I was so lucky to live in, as a PhD student at Oxford University, where the first and most crucial of my masters, Sir Michael Dummett, was also a scholar in continental metaphysics: not only Frege’s, but even Hermann Lotze, one of the German masters of Realism...

**Andrei Simionescu-Panait:** In a letter to Ingarden from 1917, Edith Stein jokes about how cumbersome her work with Husserl in editing the *Ideen* manuscripts may “force” her to attain an “absolute *epoché* in terms of waiting”. However mildly funny this might be, it betrays her conception about this phenomenological instrument, as a desired and perhaps attainable philosophical *state* of consciousness with which to proceed in philosophical endeavours. In contrast to this, Luft (2011) explains how the *epoché* is only a preliminary step in the process of reduction. Both bracketing and (eventually) applying the reduction unfold as practice, by the way of exercise, which means that we're talking about processes here and not states. How do you position yourself between these two presumably opposite takes on working in phenomenology?

**Roberta De Monticelli:** I emphatically disagree from Luft’s claim that “the reduction marks a significant rupture within Husserl’s thought and stands for the establishment of Phenomenology as a transcendental philosophy in the tradition of Kant” (Luft, 2011, p. 243). It is a most popular claim, actually marking Husserl’s traditional interpretations from the very beginning. The myth of a “Kantian”, or in *that* sense “idealistic” nature of what Husserl unhappily termed “transcendental idealism” was born with the first dissents between the master and some of his former students, such as Roman Ingarden or Edith Stein, or the so called “realist” phenomenologists of the Munich Circle. I always regarded this reading of Husserl’s thought as a tragic misunderstanding of its very crucial – and utterly anti-Kantian tenets: i) the priority of the given on the construed (“Back to the things themselves”); ii) the theory of *eide, i.e.* of those *ontological* (not just epistemic) bonds of (co)variation of possible contents which make up the very material ontology of concrete objects and states of affairs or events, grounding the “laws of essence” of different ontological regions (and, by the way, of axiological regions too: for values are a subclass of *eide*); iii) intentionality explained as an (ontologically) *internal or essential* relation, i.e. as a relation whose terms cannot subsist independently of the relation itself (the terms are, technically, non-independent parts of a whole, or “moments” of it).

Now whereas ii) explains the meaning of “idealism”, which in several passages Husserl takes as referring to his theory of *eide,* iii) might be read as an “idealistic” claim in a Kantian sense. Wrongly so. For its rationale is a rigorous definition of the first-person perspective, *as an essential feature of consciousness.* All the possible different modalities of consciousness (perceptions, beliefs and propositional attitudes, emotions and feelings, desires, decisions, drives) are different modes of presence of objects (states of affairs, events, realizable possibilities), quite obviously inseparable from the subject whom they are presented in a given perspective. Yet “inseparable from” does not mean “reducible to”: quite on the contrary, everything worth the name of *real* is (given as) *transcendent* the aspect or part of it which is present, or presently “given” to consciousness. In case of direct cognition or “original presence”, such as perception, emotion, empathy or, in short, *experience* (as opposed to representative, and even “symbolic” consciousness) this transcendence lets the object appear as an *infinite source of information*, open to active exploration and capable of disconfirming illusions, resisting to cognitive mistakes and, above all, resisting to our wishes, wills, endeavours.

In this background the meaning of the phenomenological epoché can be better clarified. Epoché is the “radical alteration of the natural attitude” consisting in “bracketing” or setting out of power (without rejecting) - the “posits” built in the natural attitude (*Ideen I*, § 31). What are these “posits”? Quite simply, the obvious, pre-reflective, automatic, sometimes wrong and often right *endorsements* of all contingent particularity of both object and subject bound in a given intentional relation, or state of consciousness. Let’s exemplify with perception. “Posit” is the tacit stance taken by the perceiver (*Stellungnahme*) as concerns the true existence of the thing perceived. Even if it can turn out wrong (in case of a perceptual illusion) it is no “judgement” or reflective statement: it definitely is “built in” the perception as such. A perceived thing is *given or experienced* as *existing here and now*, as opposed to a remembered or an imagined one. Here and now, that is, given *to me,* from exactly the point of view that *I myself* am presently and contingently taking over.

Why bracketing posits? That’s the way to “alter” the natural attitude, i.e. *to switch to the phenomenological attitude.* Bracketing all contingencies and circumstantialities in an intentional state is just how we proceed in order to regard this state *as a token of its type*, as an instance of – say – visual perception, accessible to everybody in its essential or invariant or non-contingent features (concerning both its object, its subject, and their mode of presence/givenness to each other). The point is: phenomenology is a method of research, aiming at universally accessible evidence; but it cannot achieve universal accessibility *by giving up first-person perspective*, and switching to third-person, or “impersonal” one, as Scientific Naturalism does by adopting the positive stance of an empirical science, e.g. cognitive psychology. It is specific to phenomenology to safeguard any intentional state – say perception – as it is actually lived, i.e. in first-person perspective. Living in first person perspective is (en)acting life as a subject – that’s why phenomenologists prefer the term “act” to “state” when it comes to instances of the intentional relation.

In short, *epoché* is the idealizing device by which universal accessibility of descriptions is reached, starting from actual, first person experience. It is the voluntary switch from everyday natural attitude to phenomenological attitude and *perspective.*

A brief clarification of what phenomenological perspective amounts to is in order here. It is the perspective one has when adopting the phenomenological stance via epoché. Adopting the phenomenological stance toward any object is clarifying *how that object appears* from an *appropriate* first-person perspective, e.g., a perceptual one, if it is a perceptual object, or an emotionally qualified one, if it is an object of emotional experience, and so on. To sum up: adopting the phenomenological attitude means *putting oneself ideally* in the place of the subject of some kind of intentional state. One adopts this stance “ideally” by “bracketing” whatever is contingent upon an actual object, e.g. its present situation in space and time, and upon an actual subject, e.g., this person I am.

So explained, phenomenological epoché has nothing whatsoever to do with Kantian transcendentalism (nor has it anything to do with “idealism” as opposed to “realism”). It seems to such an extent *constitutive* of the phenomenological method as such, that even where it is not explicitly theorized (as in the *Logical Investigations*) or it is qualified with caveats and distinctions (as by Scheler, Reinach, Stein, Héring and others) it is *of course* practiced as soon as the phenomenological description and analysis takes over.

Let’s come to a conclusion. I do not think that, concerning phenomenological epoché, the contrast state/process is a relevant one. I would take a stance on the side of Stein (and of Scheler) as concerns the priority of a “practical” epoché, that is of a setting out of the power of all more or less automatic *responses* to all sorts of circumstances, encounters and events, that we spontaneously enact in the life world. Some responses are further cognitive explorations, most of them are just habitual actions, life-routines like preparing breakfast or making one’s bed or submitting one’s corrected proofs to the publisher. We are constantly in action (Gallagher, 2012), this is our way to take reality seriously. Epoché as suspending, postponing action is no frivolous escape from this seriousness, but just a provisional “conversion” to philosophical reflection, aiming at a larger and deeper grasp of whatever reality we are confronted with, by setting aside automatic and pragmatic responses, often biased with prejudice and self-interested drives. This philosophical attitude can become a habit too, as Edith Stein had to acknowledge with a bitter smile. But if one reads carefully *Ideas* I, §§31-32, one will find a lot of hints to this secular and provisional withdrawal from mundane engagements which is a modern form of a monk’s habit... namely, phenomenological philosophy.

**Andrei Simionescu-Panait:** Self from surface to depth: in your (De Monticelli, 2014) schema about selfhood, you suggest a distinction bodily feelings (hunger, tiredness) and moods (anxiety, depression). The first would refer to experiencing one's body (globally felt – perhaps quantitatively) that can constitute vital values, while the second would point at the experience of one's global state (How are you? - qualitatively) that eventually gets constitutively entangled with cultural artefacts. (A) How would you respond to someone who would interpret these elements of your schema as echoes of Cartesian dualism? (B) It would be really interesting for readers to find out in what way are vital values overlapping with more abstract (not so vital) values. Architecture drawing lessons can make us perceive the “skeletons” of houses, altogether and simultaneously with the regular perception of buildings in the surrounding. Theorizing, do you think that abstract values can turn upon more those vital values and change their way of constitution in experience?

**Roberta De Monticelli:** The schema you mention is an attempt at providing a clear and systematic criterion for classifying emotional experiences within a scale representing the degree of motivational power each experience should have in a “normal” personal life. Where by “motivational power” I mean the degree of personal involvement we ascribe to an experience, or of the range/weight of decisions and choices elicited by it. Falling in love with Plato’s dialogues can have more consequences on one’s future behaviours than enjoying the pleasure of an ice cream in summertime.

This criterion exploits both poles of the intentional relation. On the “noematic” side it finds out a correlation between the height or hierarchical positions of the value-qualities envisaged by the emotional experiences (the agreeableness of an ice cream, the meaningfulness of Plato’s intellectual discoveries) and the “depth” or “centrality” in our lives of the corresponding experiences. Which is in other words the motivational power they have for us (differentiating motivational styles and hence personalities: you may be as deeply impressed by musical values as I am by political ones and vice versa). On the “noetic” side it discovers a correspondence between the layer of sensibility concerned by each (type of) experience and the layer of (one)self that the same experience “reveals”. The increasing role that each (type of) feeling plays in personal life (its motivating power, its relative degree of personal involvement) explains the deeper and deeper self-revealing power which that feeling experience exhibits/produces.

Both ideas stem from Scheler’s *Formalism;* my attempt consists in putting them together as the two terms of emotional intentionality. The result is the schematic representation of Self “from depth to surface”, i.e. the layered theory of self-constituting sensibility underlying it. By “self” I mean a person, *as she experiences herself, or as she is “given to herself”,* in that quite peculiar, irreducible way, in which anybody is not given to anybody else - that is, from a first person perspective. From this perspective, layers of sensibility *simply are layers of (one)self, as they are lived/experienced from within.*

A further step I endeavoured is that of exploiting this self-constitutive role of feeling experiences to provide a taxonomy of feeling experiences by exhibiting its ordering principle in the correspondence between the layer of sensibility concerned by each (type of) experience and the layer of (one)self that the same experience “reveals”. Not all feeling experiences, indeed, come to be felt from inside as “really” concerning oneself in one’s personal identity – one’s deepest self, so to speak. My theory suggests that *there is a specific layer of sensibility constituting individual personality,* whereas other, more basic, layers of sensibility are in a way pre-personal, i.e., both virtually shared with other animals and activated all the time from the very beginning of human life, even before a layer of well-structured personal sensibility, ordered by a personal or cultural scale of values, has developed. Call this latter the layer of sentiments, or the value-preferences ordering layer. If my theory is plausible, this is the very central, or most intimate, layer of selfhood. Call it the core-self: it is what ordinary language still calls a person’s “soul”.

This is the rationale of the partition you referred to, between sensory feelings, bodily feelings and moods on one hand, all of them belonging to the more basic pre-personal layers, “sensory” and “vital” ones; and the personal layer(s) comprising sentiments, sentiment-based emotions, passions. Incidentally, moods do indeed provide us with information about our global state, whose experience can be inclusive of bodily feelings (being sleepy, feeling oppressed, feeling tired or hungry) or not: yet they don’t really tell us “who” we are (as an outburst of indignation, a feeling of admiration, a surge of compassion or of contempt occasionally do, by revealing our actual value preferences to us and to others). Feeling experiences of the vital layer simply tell us *how* we are, or what we need.

The preceding clarification may lay out some answers to your questions. (A) No Cartesian dualism in the distinction between vital and personal layers of sensibility, because they are in fact layers of (one)self, of the same person as given to herself – as different, more or less central or deep “parts” – yet inseparable parts – of the concrete whole that a person is – an embodied person, of course: do we know of other ones? The ontologically independent being is the whole, not its parts. Hence there is no dualism of substances. (B) “Abstract” values, if we mean by that values of the personal level, can indeed reveal themselves as essentially dependent on the values of the vital level, and normally do: there is no personal flourishing in endeavors striving after justice, truth or beauty, without a minimum satisfaction of basic needs – hunger, having a shelter, security… Even if you may as well “sacrifice” your health or life to your passion for truth or for justice. And this tells us, once again, the (correctly felt) hierarchic relations of these value-spheres.

**Andrei Simionescu-Panait:** Husserlian phenomenology has intriguing concepts. Some of them are static and aim at mapping consciousness (objects, essences, evidences, or the later life world “regions”), while others try to seize the way in which consciousness processes unfold (intentionality, syntheses, constitution). Husserl is always under the tension between offering radical answers (in methodology at least) and inviting phenomenologists to ponder at the elusive character of consciousness, that simply can't stay still in order to be analysed (difficult to practice). Post-Husserlian phenomenologists borrow and reanimate the importance of some concepts from Husserl, in different combinations that constitute their own “voices” in phenomenology. It is said about Schoenberg that he was the first, the last and by far the best of serialists, because only he knew how to practice his own custom-tailored radical method. (A) In what way can phenomenology avoid this harsh but possible future evaluation? (B) Science rarely wonders about its foundations. Husserl fought to make a radical change when it comes to those heavy cornerstones of human enquiries. But this was one century ago. Now, can phenomenological practice also be conceived outside the realm of books, while fierce discussions about methodology can be tamed and put into the background (like in regular science)? Or is this a step back towards regular, “natural attitude” driven, pre-phenomenological science?

**Roberta De Monticelli:** I have always believed that phenomenology is applied phenomenology, or it’s nothing at all. Endless methodological discussions, or worse, philological ones on the true exegesis of the sacred texts – betray the very spirit of phenomenology. It’s indeed ironic that the philosopher who revived a living way of thinking based on lived experience, the one who used to ban sheer speculation or just deductive thought not supported by the intuitive *presence* of the things themselves about which one is thinking, was also a compulsive writer, one who could not think without writing. Yet Husserl is not the only classical source of our methodology and technicalities. If there is a first limit to overcome in the scholarly training of young phenomenologists, that is a too exclusive study of Husserl (or, worse, of Husserl as interpreted or criticised by Heidegger). There are plenty of other sources even among the very classics, from Scheler to Stein, from Reinach to Spiegelberg, from Pfaender to von Hildebrand, not to mention Hedwig Conrad-Martius, Gertha Walter, Nikolai Hartmann, Rudolf Plessner... But the second, even more harmful limit to overcome is a purely scholarly training, confined to reading literature without practicing phenomenological analysis and description of particular issues. Our Centre in Milan tries to improve teaching in both directions: by enlarging the “canon” – and including not only the mentioned authors but also many contemporary ones, and many “applied phenomenologists” from different disciplines: psychiatrists, psychologists, social ontologists, legal philosophers, moral philosophers. And, more importantly, by focusing on particular topics, allowing for discussion and comparison between phenomenological and other analytical approaches, and suggesting personal exercise in the techniques of eidetic variation, design of mental or empirical experiments, rewording of many classical issues in phenomenological terms: free will, personhood, personal identity, consciousness, emotions, perceptions, value theory, normativity theory, collective intentionality, intersubjectivity. All this can count as answers to your questions (A) and (B). I shall add that the best practice of phenomenology requires a training in that kind of structural-eidetic exploration, discovering “laws of essence”, which is *based on experience*, but in a way different from empirical or statistical induction: eidetic generalizations are based on *experienced tokens* of *typical or eidetic cases*. Such generalizations are *necessarily* true if they are true, and their epistemic status is *apriori*. Yet they are far from being “purely conceptual” or just rooted in semantics: they really are *substantive* relations, rooted in the contents of the envisaged states of affairs, as given to experiences of the appropriate sort.

**Andrei Simionescu-Panait:** In an article from 1999 in Zahavi, Depraz (Eds.), P. Ducat sees phenomenology as a discipline that is supposed to be separated from ideologies of any sort (Ducat, 1999). Phenomenology and miscellaneous political fashions of any intensity cannot, or at least should not suffer any form of marriage. However, the transcendental structures behind political attitudes in humans sounds like a valid phenomenological application, since it's engaged in analysing experience. What are your predictions on the way in which phenomenology can reconfigure political sciences?

**Roberta De Monticelli:** It seems to me, though, that Ducat holds phenomenology to be independent from *ideologies* of any sort, not from *ideals.* The point is that this crucial distinction is more and more fading away from the thought and consciousness of many contemporary philosophers, both on the side of Scientific Naturalism and on that of Post-modernist disenchantment. This I judge to be a tragic mistake. Even Ducat seems well conscious of that Husserlian claim on the interweaving of ethics and logics, or the practical and theoretical sides of reason, that we discussed above.

Reason can be defined as (not only) a disposition, (but also) a willingness *to respond adequately* to all sorts of exigency and demand raised by the life world. These “demands” range from the most basic and simple “affordances”, such as the aesthetic and functional qualities of items in the natural and artificial environment (which have a lot of motivational power but are no unconditional “oughts”) up to the conditional and unconditional obligations pervading the realms of morals, law and politics. Obligations and requirements pervade also, as we saw, the realms of logics and cognitive research or technology. Norms and their critical examination, issuing in either renewed, universally accessible justification, or else in their reject (at least as *universally* valid norms), are everywhere subject to what Husserl called “the jurisdiction of reason”. More than that: there is no intentional state (or rather act) which would not be subject to this “jurisdiction”. For experience and consciousness crave for adequacy. A perception can be illusory, an emotion inappropriate, a desire inconvenient, a will unjust, a judgment false, a sentiment wrong, a piece of imagination incoherent or impossible.

What is curiously neglected by the whole Brentanian and analytic tradition about intentionality is *this* “noetic” side of the intentional relation, namely the *validity claim* built into each act, depending on its characteristic “posit”, making up the naïve dogmatism of common sense, ordinary experience, traditional beliefs, shared or “social” emotions and sentiments. It is an implicit claim, spanning each act, without which we could not even discover our cognitive, emotional and practical mistakes, and “learn from experience”. Hence it is really constitutive of any intentional act, quite as much as its relation to an intentional object, despite being ignored by Brentano and the analytical tradition. Yet this validity claim can turn out to be justifiable, or not. The very possibility of *questioning* the rightness of any act whatsoever, or the critical possibility, certifies the overall “jurisdiction of reason” on personal consciousness and experience. This discovery is the act of birth of philosophy. Even according to Husserl’s rich lecture texts on history of philosophy and, what is more, human civilization (Husserl, 1956a, 1976, 1988a, 1988b).

An examined life, though, is by no means an anthropological necessity, but only a habitual, freely endorsed personal attitude, first introduced as a foundation of a civilized and just society by Socratic philosophy in the Ancient world, and by Enlightenment in the Modern world. It is an attitude massively neglected by most cultures and civilizations throughout history and geography. It is the personal attitude corresponding to what Husserl calls the Socratic and humanistic ideal of a “civilization” founded on reason (i.e. as we saw, on *demands* of reasons and justification: quite the opposite of that disquieting “panlogism” of a Hegelian “Reason” issuing in historical determinism, political realism and “ethical State”). A civilization founded on reason should be understood as opposed to a civilisation founded on tradition, religion (God’s will), or whatever “voluntarism” of the modern age might justify modern *Realpolitik.* That is, the reject of the rule of law in favour of a man’s government and of his decision-making powers, along the lines of political thought of, say, Carl Schmitt, Heidegger himself, and post-Foucaultian schools (such as the so-called Italian Theory with Agamben and others) (Esposito, Gentili, & Marramao, 2015).

This brings us back to your question. On one hand, Husserl has even a peculiar term for what I called Ideals, referring to the project of a civilization founded on reason(s). This term is “telos”, yet this word should be understood as a synonym of “task”, “duty”, overall project of our practical thought, subject to an “always renewed” verification and criticism. And by no means as a “destiny” of “western” civilisation, since it is quite the opposite of a destiny. It is just a past commitment, freely obliging autonomous moral subjects capable of verifying it and endorsing it again, maybe in new forms. It is an “*immer wieder*” rising demand of (public) justice, freely and rightly accepted and engaging one to corresponding action, or freely and wrongly rejected by delivering oneself, for example, to dark totalitarian and nationalistic adventures of the Nazi kind. This is why Husserl not only does not reject, but passionately advocates *a phenomenological foundation of political theory,* as part of a “rational science of society”, where, of course, rationality is meant in its double face, theoretical and practical (Husserl, 1956a)^ii^. More than that: Husserl has a lucid grasp – one would say a prophetic vision – of the ideal of a European Unity yet to come. Europe, much more than a continent, is a movement toward the ideal: a recursive affirmation of values over and above facts, ideas over and above reality, ideal obligations over and above actual powers. It could not have been home to science and democracy otherwise.

On the other hand, it would be quite surprising if political obligation did not find a place, definitely not reducible to, and yet conditioned by, legal and moral obligation, within the general scheme of *material ontologies* (e.g. social ontology) and *material axiology*, two main research fields of phenomenology. Between the spheres of values (of a personal level) we definitely find the sphere of political values, or values making up a well ordered polity (or a project thereof): justice, with all its “moments”, or partial contents: responsible freedom, equal dignity-and-rights, equal opportunities, minimum welfare, solidarity, citizenship, legality, transparency, pluralism, independence of the truth agencies such as information, education, judiciary... Here is a vast program of analysis and discovery for a *material axiology of phenomenological obedience*.

**Andrei Simionescu-Panait:** If we were to check correspondence from the First World War, we see that Edith Stein was very unhappy with the way Husserl gave next to no feedback on his students' text, as well with the very unstable way in which Husserl would share his thoughts on how his own Ideen manuscripts should be handled by his followers. Their communication (cooperation) was precarious because of Husserl's fast paced rhythm of writing on new manuscripts. As expected, Husserl was in turn dissatisfied of the reception of his phenomenology, dissatisfied with his students' phenomenological directions as he grew older and became dominated by his genetic attitude in thought. He was especially dissatisfied with Heidegger, in a letter to Pfaender from 1933: “I arrived at the distressing conclusion that philosophically I have nothing to do with this Heideggerian profundity, with this brilliant unscientific genius; that Heidegger’s criticism, both open and veiled, is based upon a gross misunderstanding; that he may be involved in the formation of a philosophical system of the kind which I have always considered it my life's work to make forever impossible. Everyone except me has realized this for a long time.”, Husserl (1997) writes, as he seems to take the blame for not being very attentive to his students' progress. But is he to blame? When a great and cunning philosopher fails as a teacher and as a group leader, can we attach the label of failure to his name? The question would be: can we expect from a person to be both experimental, radical, fresh in thought - and well organized and patient with pupils – or is it a conflict of possibilities in terms of attitudes and values?

**Roberta De Monticelli:** We are back to the misunderstandings concerning the real legacy of phenomenology, but in the context of a larger, interesting inquiry into philosophical pedagogy. Let me come back to Husserl-Heidegger first, this time with a personal experience. Husserl’s *Krisis* (Husserl, 1976) was the first text in classical phenomenology I read – even before taking up philosophy at the University. Enzo Paci, the founder of Milan’s phenomenological school (which was somehow dispersed in its rich potentialities, owing to the somewhat eclectic character of Paci’s thought: yet on that far background of our Centre, see De Monticelli, 2011) was then very popular, even school pupils and cultivated people had heard of him and of the translations he fostered of Husserl’s masterpieces. I was a 16 years old girl when 1968 exploded, and I remember feeling perfectly at ease with that confused mixture of Frankfurter “critical theory”, Hegelianism, and undigested bits of phenomenology, which was a sort of common language for the young leftists of those days. It was only thirty years later that, going back to the *Krisis* to prepare an undergraduate course, I was almost shocked by Paci’s Preface of 1968. *Not a single word* about the tragic time in which Husserl had written the first seven sections of the *Krisis,* where he manages to condense his thought by presenting it in direct continuity with modern Enlightenment, both nourished by the hope to provide at last a foundation of *knowledge*, *reasonableness* and *autonomy* to practical thought (ethics, legal and political theory). Instead of accepting the gap between science, which does admit of such a foundation, and the governance of human affairs, left over to arbitrariness, *Realpolitik*, or the conflict of faiths. *Not a single word* about the tragic meaning of Husserl picture of philosophers as “Funktionäre der Menschheit”, officials of humanity – as written by a man who had just been deprived of his role of public official, that is of his *venia legendi,* the power to teach, by his own university, with the approval of his former student, Martin Heidegger. *Not a single word* about the two principles affirmed throughout those seven sections. That is, I) the principle of responsible personhood, grounding practical reason on freedom of the moral subject, conceived as a reasonable, sensible and autonomous agent, capable of *value experience and research,* and II) the principle of moral, legal and cosmopolitical universalism, grounded on universal accessibility of the fundamentals of public ethics, making up what post-war humanity called “the human rights”.

*Not a single word* on the fact that Heidegger had built his “system of thought”, in those years, *on the very reject of those two principles*, and on their replacement with two opposite principles. Here they are: I) a principle of historical destiny, removing autonomy and moral responsibility of individual persons; and II) a principle of ethnic, *Blut-und-Boden* community as prior to that of personhood and equal dignity, on the basis of which he enslaved philosophy to the exaltation of the *Fuehrerprinzip,* and of the “intimate greatness of national socialism” (*Rectorship Speech*).

In this respect, compared with some masters of my generation, even the celebrated (or ill-famed) kind of solipsism of Husserl’s teaching was a paradigm of rigour, intellectual honesty and passion for truth. As one gathers, after all, from the memories of Edith Stein, Ingarden, Spiegelberg and many others – all of them recognizing, as a defining mark of true phenomenology, that crucial love for “*synphilosophein*”, or the debating community of philosophers, preferring attention to “the thing itself” and to each other’s work, to monologue in later “Hirsch-of-Being’s style”. After all the dream of reviving an international community of researchers, truth being “an ideal situated in infinity” and not the revelation of some guru or visionary bard, is a recurring dream of phenomenologists of all ages. Our Centre too is still dreaming that dream, and you and I are collaborating to make it more real, in this very moment.

**Andrei Simionescu-Panait:** You have had a productive publishing rhythm in recent years. *Ontologia del nuovo: la rivoluzione fenomenologica e la ricerca oggi* (2008), *La novità di ognuno. Persona e libertà* (2009) or *L'ordine del cuore. Etica e teoria del sentire* (2012) are some of your works in recent years, in which you analysed the manners in which phenomenology today can reshape ethics, epistemology, or questions about selfhood and choice, to name a few. Towards which area of human experience will your next project be focused?

**Roberta De Monticelli:** Let me just add the last group of works on moral and civil issues in contemporary Italy, *La questione morale* (2010), *La questione civile* (2011) and *Sull’idea di rinnovamento* (2013), if only to stress once again the link I feel mandatory for a Socratic philosopher, between pure research and participation in public debate. I tried to perform this duty by taking a stance in the “space of reasons”, or in the face to face of argument and criticism, against a tendency of governments to bypass legality and even constitutional norms in favour of informal powers and particular constituted interests. Using the empirical materials of some battles against corruption or lack of transparency in the public sphere I tried a phenomenological investigation of our so widespread indifference to public ethics, finding its origin in a special kind of self-destitution of the autonomous moral subject, in a cultural climate of moral and political scepticism. All this descriptive materials form the background and intuitive source of evidence for an outline of a phenomenological theory of values, opposing most meta-ethical positions, from metaphysical realism to expressivism, emotivism, constructivism of different sorts. The result is now in print – it will come out next Fall under the anti-Nietzschean title *Al di qua del bene e del male* (Einaudi).

Yet my research project for the future is different. I have written even too much in Italian or French, in an age where you should not escape confrontation and criticism on the part of the international philosophical community. I’m writing in English (or a faint imitation of it) several papers and the project of a book tackling with what I deem to be a real gap in the current phenomenological renaissance: the theory of ontological dependencies, i.e., eidetic invariants and “the gift of bonds”. My utopian dream is that of rethinking that sort of Ockham’s fork which set modern thought on the path of naturalistic metaphysics, empiricist epistemology and axiological scepticism. I am trying to think anew, by contemporary standards of rigour and exactness, the other path – the one whose rejection threw back Duns Scotus and the essentialistic tradition among the myths or the fairy tales of the past. Wrongly so – for it is the path of phenomenology, conceived as *an ontology of concreteness.* Some initial hints at this research program are to be found in De Monticelli (2012) and De Monticelli (in press).

**Andrei Simionescu-Panait:** One last question, a curiosity. You're known to have a close relationship to literature, novels and poetry, and it seems that the best of authors manage to informally capture specific particularities about human attitudes, situations and changes that blend all of these together. They can seize the shapes of flowing experience, they can grasp contrasting emotions in events and successful recurrences of failure in characters, they can see “essences” in motion. I wonder, which author in literature would you consider to have great phenomenological intuitions, even if lacking formal phenomenological training?

**Roberta De Monticelli:** There is a question that has always been intriguing to me: how comes that we learn much more about the essential features of emotional experiences by reading novels, poems or ancient and modern tragedy and comedy, rather than by studying dedicated chapters in psychology handbooks? I think I found an answer to this quandary. Emotional phenomena are dependent not only on personhood, but even on individual personality, because they are *constitutive* of both. They are both individuating and individuated! Quite as much as actions, where “*si duo faciunt non est unum*”, the same type of action is instantiated by essentially different tokens depending on the agent. Recall the dramatic differences in the ways people have to walk, to speak, to respond to similar circumstances. Conversely, one will find this person’s voice, dynamic style, smile, unmistakable or unique. The same happens with emotions and sentiments of a personal level. They don’t just manifest personhood, but individual personality. In the very richness of their contents and implications, they cannot be separated from the individual person experiencing and enacting – somehow “interpreting” them. Think of the deep differences between the ways different familiar persons have to be and to appear unhappy. This is *not* the case with instances of logical or mathematical reasoning, or even philosophical *thoughts*, that, as Frege used to say, “have no master”. And in fact, as far as psychology of cognition, logics or even perception is concerned, we do learn from academic psychology much more than from literary texts. Acts of the strictly cognitive spheres are *not* individuated and individuating.

A phenomenologist (e.g.: Edith Stein, 2014) would say: only emotional and conative acts, in short being affected and acting are self-constitutive acts, or experiences through which we “encounter” ourselves, or the others, in their intimate individuality. Purely cognitive acts are not self-constitutive, I can be completely unaware of myself when thinking or calculating (but try to be unaware of yourself while somebody steps on your foot).

That’s why we seek to understand different types of ill-fated love, perhaps, by reading Anna Karenina or Madame Bovary (that is, in the background of a personal narrative, as many would say) much more than by studying academic psychology.

As a way of conclusion, I would say that any great novelist or poet is a master in phenomenology of *haecceity* – of personality or essential individuality of persons, as it unfolds through all value and disvalue experiences of a given life, confronting good and evil of all sorts. A character, in this sense, is very much as you say, “essence in motion”. We have often more vivid memories of fictional characters, encountered in literary worlds, than of some real persons met in our environment. What young girl would not fall in love with Prince Andrei Bolkonsky on a ball night in Moscow, exactly like Natasha?
